# Atomic Force Microscopy Crosslinks Interdisciplinary Eye Research

**Published:** 2015

**Authors:** Christian M. Hammer, Tilman E. Schäffer

**Affiliations:** 1 Department of Anatomy II, Friedrich-Alexander-University, Erlangen, Germany; 2 Department of Ophthalmology, Friedrich-Alexander-University, Erlangen, Germany; 3 Department of Applied Physics and LISA+, Eberhard-Karls-University, Tübingen, Germany

**Keywords:** Atomic Force Microscopy, Eye Disease, Interdisciplinary Eye Research

## Introduction

Over the past decades, it has always been a pleasure to see how the field of medical research unites with other areas of scientific expertise for the sake of medical progress. From these connections, groundbreaking beneficial devices and applications like computed axial tomography (CAT) or magnetic resonance imaging (MRI) have sprung (1, 2).

In the ophthalmology departments and eye clinics, it has as well become almost impossible to imagine life without optical coherence tomography (OCT), surgical lasers and a variety of other techniques. Their constant refinements and upgrades by medical and natural scientists working together make them even more effective and indispensable in ophthalmologic practice.

In the examples given here, medical engineers and physicists play the main roles, working hand in hand with physicians and medical researchers, to invent new diagnostic tools and therapeutic strategies.

For the ophthalmological research, giant leaps forward are often accomplished when physics departments or facilities and institutions of classical eye-related research share promising projects and common hypotheses. Ocular biomechanics is a wonderful example of a research area defined by both biomedical and physical aspects of normal and pathologically altered ocular tissues.

**Figure 1:**
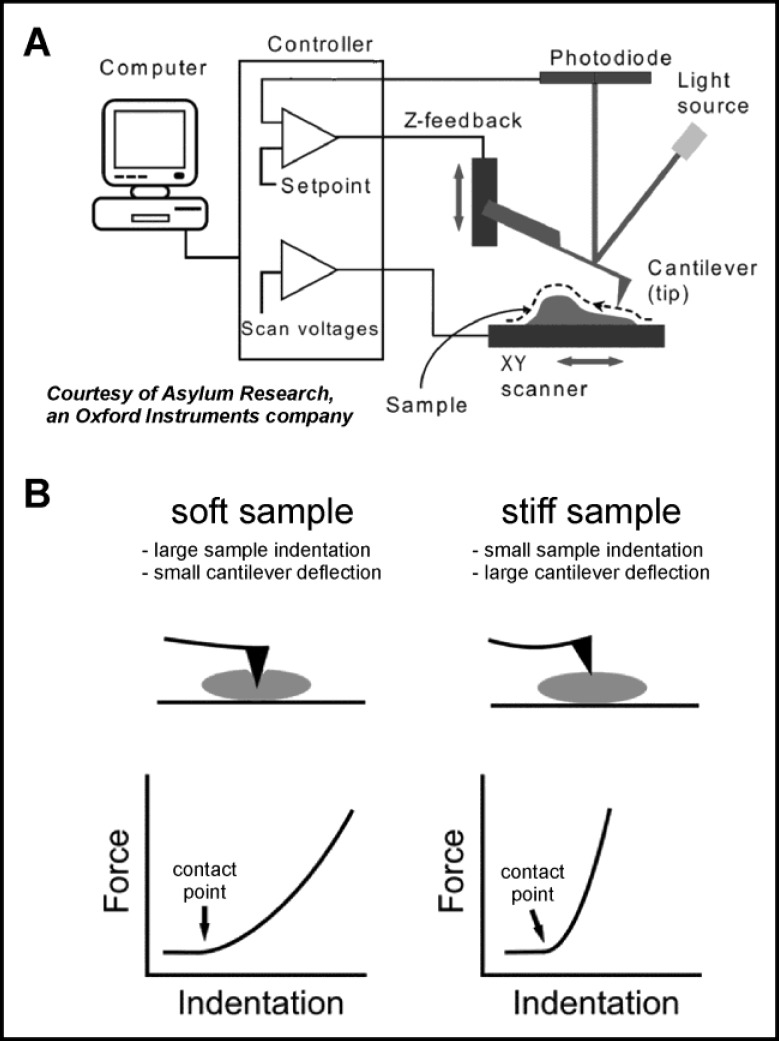
Simplified schematic of the setup of an atomic force microscope (with kind permission of Asylum Research, Santa Barbara, CA, USA). The topography of the sample is scanned with a cantilever tip and recorded by a computer. Cantilever deflection is measured with a laser beam reflected from the cantilever’s back. B: Principle of nanoindentation measurement. If the cantilever is pressed upon a soft sample (left), it will markedly indent the sample, but deflect only moderately. The converse is the case with a stiff sample (right).

In the past, uniaxial and biaxial stress-strain measurements were used to biomechanically characterize the viscoelasticity of the posterior sclera near the optic nerve head (3,4). This region, called peripapillary sclera, is assumed to be of critical importance in the development of glaucoma. As a matter of fact, nearby tissue, the lamina cribrosa, is actually subjected to the most pronounced morphological alterations associated with glaucoma-induced blindness. For at least one glaucoma subtype, the so-called pseudo-exfoliation (PEX) glaucoma, theories postulate a preceding history of defective connective tissue metabolism, manifesting itself as PEX syndrome (5). There is a growing body of evidence that this may render the lamina cribrosa structurally weakened and, hence, susceptible to pathological outward distortion of the optic disc, driven by the always present intraocular pressure (optic nerve head cupping). As the lamina cribrosa looks like a porous and elastic plate scaffolding for the retinal ganglion cell axons when they leave the eyeball, it is easy to imagine that its excessive and prolonged distortion might impart severe stress on these cells. This may damage and ultimately kill the retinal ganglion cells and result in blindness. The problem with the lamina cribrosa is, however, that it is very difficult to dissect, isolate and to subject to conclusive stress-strain measurements. Thus, in order to test whether the connective tissue of the lamina cribrosa is really weakened in PEX syndrome, alternative approaches had to be found. An elegant solution was to employ atomic force microscopy (AFM) to measure the Young’s modulus of the lamina cribrosa tissue beams (6). Essentially, an atomic force microscope can be used to image a sample’s surface topography and to characterize a sample’s biomechanical properties. Central element of an atomic force microscope is a cantilever endowed with a cantilever tip (looking similar to a record player needle). A laser beam is reflected from the back of the cantilever and detected by a position-sensitive photodiode. If the cantilever tip is scanned across a sample, the cantilever will be deflected to a higher or lower degree, depending on the local sample height (Fig. 1A). The higher the sample at a defined location, the larger the cantilever deflection and, hence, the laser beam displacement on the diode. From this, the surface topography of the sample can be computed. Furthermore, AFM can be used to indent tissue on a microscopic scale with the cantilever tip (Fig. 1B). If the tissue sample is comparatively soft, pushing the tip into the sample will easily indent it, while the cantilever deflection will be small. Stiffer tissues, however, will cause the cantilever to deflect substantially, while tissue indentation is decreased. The indentation force is calculated from the deflection using the spring constant of the cantilever. From the force vs. indentation relationship, the local Young’s modulus can be determined by fitting the Hertz-model (6, 7). Reiterating this procedure within the region of interest in a grid pattern is called force mapping, which gives an image of sample stiffness (quantified as Young’s modulus).

We applied force mapping to the lamina cribrosa of three deceased PEX syndrome patients and three normal controls. We were able to selectively image and biomechanically test the lamina cribrosa connective tissue beams, ignoring pores filled with neural tissue. We also analyzed the peripapillary sclera correspondingly. This sophisticated method yielded results that were consistent with the aforementioned theory. For the first time, it was possible to show that the average Young’s modules of a PEX group were significantly decreased in both the lamina cribrosa and peripapillary sclera. Thus, combining the expertise in advanced AFM application (Schäffer TE et al.) with that in ophthalmology (Hammer CM et al.), allowed us to disentangle a problem that had bothered glaucoma specialists for a long time.

While strengthening this interdisciplinary approach, we focused our attention on a very different ophthalmological problem related to corneal biomechanics -- the keratoconus. Keratoconus is a refractive defect of the eye caused by a substantially weakened and/or thinned central cornea (8). Due to the intraocular pressure, this region bulges outward and gives rise to a cone-shaped form of corneal ectasia. This defect normally develops progressively, over years, and results in an increasing refractive error, difficult to correct with glasses or contact lenses. However, if diagnosed early, keratoconus progression can be arrested and partially reversed by a minimally invasive intervention called corneal collagen crosslinking (CXL) (8, 9).

In the standard procedure, the corneal epithelium is abraded to undo its barrier function. Then, the exposed stroma is imbibed with the photosensitizer riboflavin (vitamin B2) and subsequently irradiated with UVA light (370 nm). This induces the formation of collagen crosslinks, which increases the intrinsic biomechanical strength of the cornea. In the past, stress-strain measurements were utilized successfully to prove the CXL-induced augmentation of the corneal Young’s modulus (10). However, it has been unclear for a long time how deep the crosslinking effect reaches into the stroma. Due to the depth-dependent and riboflavin-enhanced absorption of the UVA radiation scientists assumed that effective crosslinking was restricted to the anterior stroma. Yet again, stress-strain measurements gave first experimental evidence verifying this hypothesis. Kohlhaas et al. used a microkeratome to cut two sequential stromal lamellae of 200 µm thickness in cross-linked and non-cross-linked porcine and human eyes (11). Only the anterior lamella was found to display a significant increase in corneal strength, whereas the posterior lamella showed no difference from the controls. From this, it was assumed that effective CXL was confined to the anterior 200 µm of the corneal stroma. However, only a continuous depth-dependent profile of Young’s modulus would have allowed for an accurate determination and characterization of the zone exhibiting effective CXL. This was exactly the gap we were able to fill with a modified version of our AFM force mapping technique (7). Several regions of interest were placed threaded together, collectively spanning the entire depth of the central corneal stroma.

This way, we were able to generate the first continuous depth profile of corneal Young’s modulus after CXL with a mechanical probe. In that vein, we verified the findings of Kohlhaas et al., localizing the maximum depth of effective CXL at 220 µm. This confirmed previous estimates of other scientists who predicted the maximum depth of the CXL zone to be spotted between 200 µm and 300 µm (11-13). Moreover, our data demonstrated an exponential decrease of Young’s modulus with increasing depth, which is in line with Schumacher et al. who showed the intensity of UVA light to decline exponentially with increasing stromal depth (13).

With the two examples of interdisciplinary eye research given, we would like to emphasize the immense potential inherent in AFM force mapping and interdisciplinary research for the biomedical sciences in general and for ophthalmological research in particular. If researchers of different areas of expertise continue to find common hypotheses and a common language to communicate ideas, problems and methodological limitations, chances are high for a successful outcome. Besides, we are convinced that venturing into an initially unfamiliar scientific field and integrating parts of it into one’s own research area tremendously enriches and widens the academic scope of one’s mindset. This, of course, is not only of value for scientific progress, but also for personal development. A pivotal prerequisite for this, however, is a certain degree of interest and respect for each collaborator’s scientific field. Moreover, a mutual account of and tolerance for a collaborator’s partial lack of knowledge pertaining to one’s own area of expertise is essential. Therefore, a minimum of open-mindedness is required, among others, to work hand in hand across the scientific disciplines. We, for our part, have found interdisciplinary research in the field of ophthalmology both scientifically fruitful and intellectually enriching. We can only recommend it.
